# Effects of Long-Term Freeze–Thaw Cycles on the Properties of Stabilized/Solidified Lead-Zinc-Cadmium Composite-Contaminated Soil

**DOI:** 10.3390/ijerph18116114

**Published:** 2021-06-06

**Authors:** Zhongping Yang, Jiazhuo Chang, Yao Wang, Xuyong Li, Shu Li

**Affiliations:** 1 School of Civil Engineering, Chongqing University, Chongqing 400045, China; Alongdesk@163.com (J.C.); a_yao0218@163.com (Y.W.); lixuyong@cqu.edu.cn (X.L.); 2 Key Laboratory of New Technology for Construction of Cities in Mountain Area, Chongqing University, Ministry of Education, Chongqing 400045, China; 3 National Joint Engineering Research Centre for Prevention and Control of Environmental Geological Hazards in the TGR Area, Chongqing University, Chongqing 400045, China; 4 Chongqing Geotechnical Engineering Testing Centre Co., Ltd., Chongqing 400045, China; cqytjc2013@163.com

**Keywords:** long-term freeze–thaw cycles, composite heavy metal contamination, solidification/stabilization, mechanical properties, environmental influence, micro-mechanism

## Abstract

Lead, zinc, and cadmium were used to prepare a composite-contaminated soil to replicate common situations, in which soil is usually simultaneously contaminated by multiple metals. To examine the long-term durability of stabilized/solidified (S/S) contaminated soil, specimens were subjected to a series of freeze–thaw (F-T) cycles, up to ninety times (one day per cycle), prior to testing. Triaxial compression tests, soil column leaching tests, and X-ray diffraction analysis were then employed to study the mechanical properties, environmental influences, and micro-mechanisms of the S/S lead-zinc-cadmium composite-contaminated soils after long-term F-T. The results showed that triaxial compressive strength increases within three F-T cycles, then decreases before slightly increasing or stabilizing after thirty F-T cycles. The stage of decreased cohesion thus occurs between three and fourteen F-T cycles, with variation in other factors similar to that of the triaxial compressive strength. The cohesion mainly increases between three and seven cycles. The soil column leaching test showed that the permeability of soil is more than four times higher than that of soil not subject to freeze–thaw cycles after ninety F-T cycles. XRD tests further revealed that the chemical composition of S/S contaminated soil and the occurrence of each heavy metal (HM) remained unchanged under F-T treatment.

## 1. Introduction

The ongoing development of world industry has brought great damage to the soil environment, especially in terms of the impact of heavy metal pollution [1,2,3,4]. According to World Bank statistics, the world’s industrial added value has been rising since 2010, and the number of countries in which industrial added value accounts for more than a quarter of the GDP reached 107 by 2019. This extensive development of industry has thus caused the heavy metal pollution of soil to become one of the foremost environmental emergencies in the world [5,6,7,8]. Industrial heavy metal pollution is characterized by the formation of high concentrations of pollution in an initially small number of areas, followed by the expansion of polluted areas due to diffusion behaviors. The non-degradable nature of heavy metals means that concentrations of these in polluted areas generally remain high once pollution occurs, and the soil content of lead, zinc, cadmium, and other major heavy metal pollutants is often up to thousands of times higher than the environmental standard limits in areas with heavy industry [9,10,11]. The risk of heavy metal pollution is thus serious and ongoing.

The basic properties of soil may also change because of the presence of heavy metals, and construction safety issues caused by the influence of heavy metals on soil mechanical properties have been reported worldwide [9,12]. Additionally, deeper concerns have been triggered by the fact that soil heavy metals could spread to other spheres, such as the hydrosphere, atmosphere, anthroposphere, and biosphere, due to the intimate connections between these environments, thus posing extensive and long-lasting threats to both the ecosystem and human health [13,14,15,16,17]. To help ensure the safety of human, animal, and plant life alongside the safety of building projects, it is thus crucial to carry out effective remediation works with regard to heavy metal-contaminated soil.

Solidification/stabilization (S/S) technology is widely used in the treatment of heavy metal-contaminated soil due to its good remediation effects, operational convenience, and cost-efficiency [2,18,19,20]. By adding binders to contaminated soil, S/S technology facilitates the improvement of various soil properties and minimizes pollutants’ environmental impacts by reducing soil porosity, increasing soil strength, and immobilizing heavy metal ions, which is achieved by utilizing adsorption, precipitation, and encapsulation [12,21,22].

Stabilized/solidified soil is eventually returned to the natural environment, where its mechanical properties and pollutant fixation must thus be tested. Various studies have shown that external forces, such as the freeze–thaw (F-T) cycle [23,24,25], carbonization [26,27,28], the dry–wet cycle [29,30,31], acid rain leaching [32,33,34], high alkalinity [35], and high salt groundwater infiltration [36], can affect the properties of S/S contaminated soil. Among these, F-T cycles are notable due to changing the soil’s compressive strength, permeability, and heavy metal leachability by amending the porosity ratio, crack width, and particle size of the soil, thus affecting the stability and safety of superstructures created upon it [37,38,39]. Globally, the area affected by the F-T cycle is quite large. In the Northern Hemisphere, seasonally frozen ground covers about one fifth of the total land area, and this region is expanding due to the degradation of permafrost caused by global warming, supporting a trend towards seasonally frozen soil [40,41]. Intensive studies of the effect of F-T cycles on S/S heavy metal-contaminated soil are thus warranted.

Zhou et al. [42] highlighted that various mechanical properties, such as the strength and stiffness of frozen loess, gradually weaken with an increase in the number of freeze–thaw cycles within a certain range. Further, according to Wei et al. [43], the longer each freeze–thaw cycle lasts, the more obvious the disintegration effect on soil, and the greater the quality loss of such soil. Zha et al. [44] found that freeze–thaw cycles significantly reduce soil strength, as well as determining that the greater the number of freeze–thaw cycles, the greater the extent of such reduction, while Liu et al. [45] pointed out that the number of boundary pores and internal pores in the soil also increased with an increase in the number of F-T cycles. He et al. [46] similarly showed that the soil strength decreases as the number of the F-T cycles increases, and that the porosity is positively correlated with the number of freeze–thaw cycles, based on their work with cement and lime stabilized/solidified hexavalent chromium-contaminated soil. Yang et al. [47] studied the influential properties of S/S lead-contaminated soil to determine that there are some differences in the properties of treated soils between those subjected to the long-term F-T cycle and the short-term F-T cycle.

Based on the previous research, the effect of F-T cycles on pollution effects in S/S heavy metal-contaminated soil is closely related to the length of F-T duration. Nevertheless, most of the available studies have focused on the influence of short-term F-T cycles, while according to relevant specifications, the design life of most projects is measured in decades. These studies examining only the influence of short-term F-T cycles thus reflect actual need very poorly, and it is therefore necessary to study the properties of S/S heavy metal-contaminated soil under long-term F-T conditions.

Previous research has generally been conducted on a single type of heavy metal contamination in soil, which lacks consideration of the actual situation in industry sites, where soil is usually simultaneously contaminated by multiple metals [11,48,49]. The remediation of composite heavy metal-contaminated soil is more difficult than the remediation of single heavy metal contamination, and the remediation effects may be less obvious, particularly where certain binders cannot be used due to the properties of a particular heavy metal. Competition among ions may also result in a significant decrease in the S/S effect of some ions. 

In this study, the soils used were thus spiked simultaneously with lead, zinc, and cadmium. These metals were chosen as lead, zinc, and cadmium are three of the most common pollutants in contaminated sites and can all do great harm to human health due to their high toxicity and enrichment ability [50,51,52,53]. Subsequently, the prepared samples were subjected to freezing and thawing of up to 90 cycles to examine the ongoing durability of S/S contaminated soil under long-term F-T conditions. The main objectives of this trial were to (1) obtain the effects of a long-term freezing–thawing cycle on the mechanical properties of such soil, (2) to obtain the effects of a long-term freezing–thawing cycle on the environmental influence of such soil, and (3) to obtain the effects of freeze–thaw cycles on the micro-phenomenon within such soils.

## 2. Materials and Methods

### 2.1. Material

#### 2.1.1. Soil

**Figure 1:**
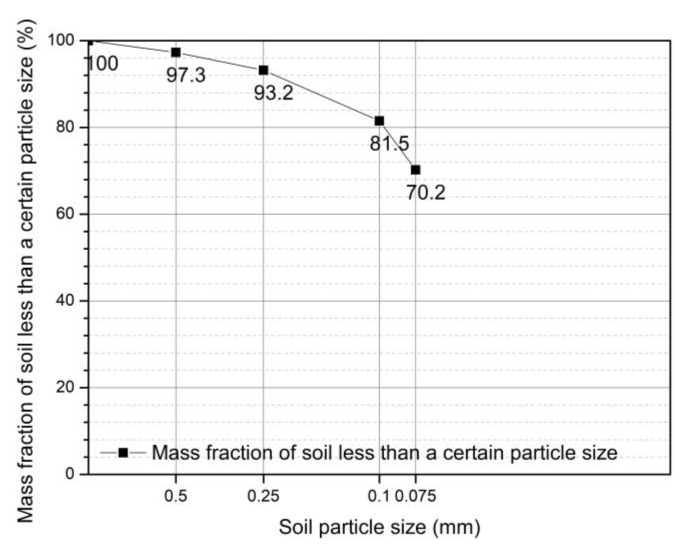
Particle grading curve.

**Figure 2:**
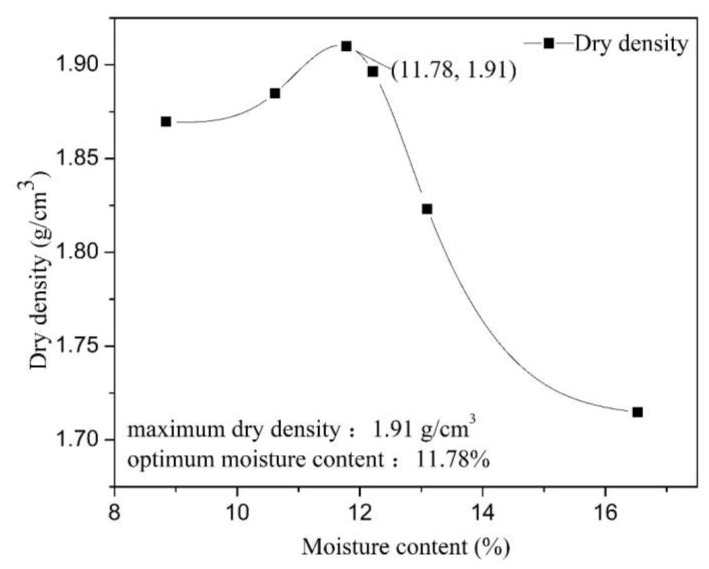
Compaction curve of undisturbed soil.

In this study, undisturbed soils of a reddish-brown hue were removed from a construction site in Gaomiao Village, Chongqing City, southwestern China. The surface soils with a thickness of about 2 cm were removed due to a lot of impurities, and soils with stable properties at the bottom were taken as the experimental soil. The in-situ soils were moist, dense, sticky to touch, and the soil quality was more uniform. There was less industrial waste, and the main debris were plant roots and rocks. These soils were dried using an electric constant temperature air-blast drying box at 100 °C for 24 h. Impurities such as various stones, grass roots, and tree roots were then removed from the dried soil, and the soil particles were crushed sufficiently to pass through a 1 mm sieve. The sieving curve of soils less than 1 mm is shown in Figure 1. The soils were stored in sealed plastic bags, and based on the Standards for Geotechnical Test Methods (GB/T 50123-2019), conventional geotechnical tests such as the limit moisture content test and the compaction test were performed to obtain the physical property parameters of the soil, which are shown in Table 1 and Figure 2. According to the Standards for Engineering classification for soil (GB/T 50145-2007), the type of soil is a low-liquid limit clay in fine-grained soil, thus, the type of soil is lean clay according to the Standard Practice for Classification of Soils for Engineering Purposes (Unified Soil Classification System) (ASTM D2487-17). X-ray fluorescence analyses were carried out on the sieved soil to identify its chemical composition and the relative content of the various substances forming the soil (as shown in Table 2).

#### 2.1.2. Binders

Lime, fly ash, and cement were selected as simultaneous additives for these experiments. The use of cement on its own requires greater energy consumption and increases carbon dioxide emissions [54,55,56]; adding appropriate amounts of lime and fly ash thus not only reduces the energy consumption, but also encourages the active substances to react with the cement to improve the solidification effect on heavy metals [57]. This also reduces the pressure to treat industrial waste fly ash, and the treatment of fly ash is an important issue with regard to industrial by-products [58,59,60,61,62,63]. The main component of quicklime is analytical-grade calcium oxide, while here, the cement was ordinary Portland cement (OPC325). The fly ash was taken from the Chongqing power plant, and the grade was determined as two according to GB/T 1594-2017. All three binders were passed through a 0.5 mm sieve to enforce uniformity, and the chemical composition and content of the three binders were obtained by X-ray fluorescence analysis, as shown in Table 3. Cement and lime usually have alkaline properties, while both acid and alkaline fly ash exist. According to the results of the X-ray fluorescence analysis, this fly ash contained the acidic oxides SiO_2_, Al_2_O_3_, and SO_3_, with SiO_2_ and Al_2_O_3_ being the activating components and playing a positive role in the solidification of heavy metals [64]. The content of SO_3_ was low, and the pH of fly ash itself does not need to be considered.

#### 2.1.3. Heavy Metals

Three heavy metals: zinc, lead, and cadmium, were used as the pollution elements in this study. As nitrate is a relatively stable substance and harmful reactions occur in stabilized/solidified contaminated soils only when nitrate concentrations are high enough [65,66], analytical-grade Zn(NO_3_)_2_, analytical-grade Pb(NO_3_)_2_, and high-purity Cd(NO_3_)_2_ were selected as the heavy metal contaminants.

### 2.2. Specimen Preparation

For these experiments, Pb(NO_3_)_2_, Zn(NO_3_)_2_, and Cd(NO_3_)_2_ were mixed with deionized water in advance. The mass ratios of Pb^2+^, Zn^2+^, and Cd^2+^ to dry soil were 8000, 5000, and 400 mg/kg, respectively. The solution was mixed, then placed in a magnetic blender and stirred for 10 min. The heavy metal solution was then added to the dry soil and stirred with a dough mixer for 10 min. After this, the soil was sealed in the moistening box for 30 days to develop artificial composite HM-contaminated soil. The temperature of the moistening box was 22 °C, and the relative humidity was 95%.

The dosages of cement (5% by weight of the soil), lime (2.5% by weight of the soil), and fly ash (2.5% by weight of the soil) were set by relative mass, and the proportions of the three curing agents were selected to develop a better mixture for stabilizing/solidifying the HM-contaminated soil based on previous research on this topic [64]. The three binders were put into the artificial composite heavy metal-contaminated soil simultaneously, and the resulting mix was stirred in a dough mixer for 10 min. The resulting soil was then sealed with a plastic membrane and kept for 24 h in a moistening box (22 °C, 95% relative humidity).

In order to obtain the mechanical properties of the soil under optimal conditions, an appropriate amount of water was added to the soil to cause soil water content to reach an optimal level (as shown in Table 1) [67].

The samples were created using the disturbed soil dry pile method. Each sample was filled five times, with the soil weight of each filling calculated using Formula (1), the sample was then weighed, and a compactor used to compact the soil until the desired compactness was achieved.

(1) m = ρV

where m is the soil weight to be weighed (g), ρ is 95% maximum dry density of soil (g/cm^3^), and V is one-fifth of the sample volume (cm^3^).

The specimens for the triaxial compression experiments were cylinders with a diameter of 39.1 mm and a height of 80 mm., and the specimens for the column leaching tests were cylinders with a diameter of 50 mm and a height of 200 mm. All created samples were placed in a standard curing chamber for 56 days, along with the remaining stabilized/solidified heavy metal-contaminated soil.

**Figure 3:**
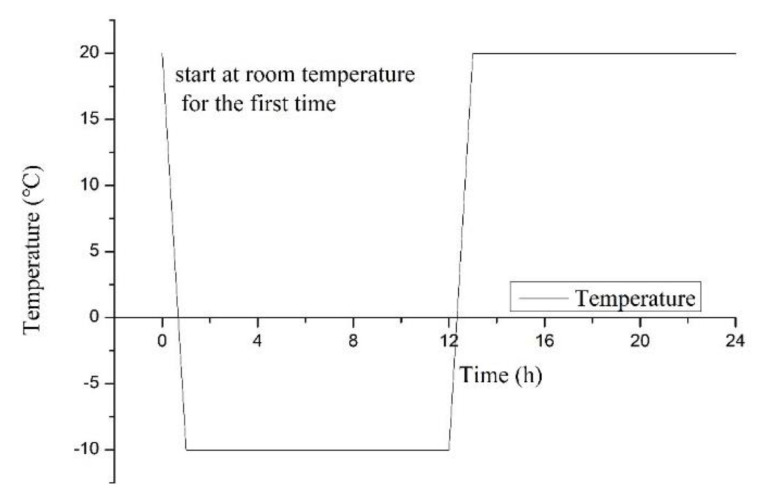
Temperature curve of a F-T cycle.

After curing for 56 days, all soils and specimens were put into the alternately high- and low-temperature test chamber (Chongqing Tester Experimental Instrument Co., Ltd.) for the freeze–thaw tests. The designated freeze–thaw cycles occurred 3, 7, 14, 30, and 90 times, with the samples without F-T cycles set as the control group. Each freeze–thaw cycle had a freezing temperature of −10 °C and a thawing temperature of 20 °C [68]. The chamber was lowered from 20 °C, or room temperature (first cooling), to −10 °C over just one hour, and it was held there for 11 h. The chamber was then heated up again to 20 °C within a further hour and remained there for 11 h, to form a full day. The periodic diagram is shown in Figure 3. This research focused on the number of freeze–thaw cycles overall, without considering the effects of different intensities.

### 2.3. Testing Methods

#### 2.3.1. Triaxial Compression Testing

Consolidation undrained tests were adopted in the experiments, and the experimental instrument utilized was a British GDS environmental geotechnical automatic permeation instrument. Three confining pressure levels (100, 200, and 300 kPa) were used, with the shear strain rate set at 0.08% per minute. When the axial strain reached 20%, the shear was stopped, and the test stopped. The size of the soil samples was then ∅39.1 × 80 mm.

Based on the results of tests on samples under different confining pressures, three limit stress circles were drawn to obtain the common tangent line, which represents the shear strength index of the soil (cohesive force, C, and internal friction angle, φ). The equation for this is as follows:

(2) $$\frac{\sigma_{1}-\sigma_{3}}{2}=C\cos\varphi -\frac{\sigma_{1}+\sigma_{3}}{2}\sin\varphi$$

where $\sigma_{1}$ is the maximum principal stress (kPa), $\sigma_{3}$ is the minimum principal stress, which is the confining stress in this study (kPa), C is the cohesive force of the soil (kPa), and φ is the internal friction angle of the soil (degree) [69].

A set of results was obtained for soil damage intensity under two types of confining pressures, each initially based on the averaged results of three sets. However, where the error of any one set was greater than 5%, only the other two groups were averaged.

#### 2.3.2. Soil Column Leaching Test

**Figure 4:**
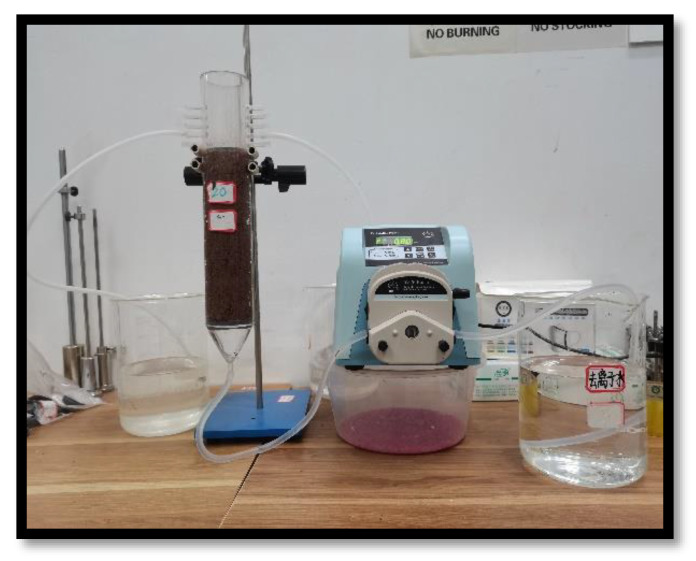
Experimental equipment for soil column leaching test.

The samples for this process were 5 cm in diameter and 20 cm in height, with the soil column held in a retainer made of PVC material. The upper and lower ends of the soil column were left open, while the lower end was slightly conical to facilitate the flow of liquid. Branches on the left and right sides of the upper end ensured that the water height remained unchanged, and that the water pressure was constant (see Figure 4).

Samples were saturated with deionized water to remove all of the air prior to the experiments. After the samples were saturated, a D100 easy-install peristaltic pump (Shanghai Huxi Analytical Instrument Factory Co., Ltd.) was used to supply water at a constant speed. A tracer element of 0.1 mol/L potassium bromide was also added.

Once the lower end of the soil column was exuding water steadily, the outflow liquid was collected. Based on the change in the magnitude of change in outflow liquid concentration, the water collection interval was then changed, however. Eventually, when the bromide ion concentration in the outflow liquid fluctuated around 0.1 mol/L, the experiment was ended. The longitudinal migration coefficient (D) was then obtained after data processing.

Taylor et al. [70,71] explained the coupling properties between convection, diffusion, and chemical reactions, establishing the relevant convection–dispersion equation. For the soil column composed of homogeneous soil, under stable and low-flow velocity field conditions, as applied in this paper, the “three-point formula” for solving for the solute longitudinal migration coefficient of saturated soil is:

(3) $$D=\frac{v^{2}}{8t_{0.5}}{\left(t_{0.84}-t_{0.16}\right)}^{2}$$

where $t_{0.16}$, $t_{0.5}$, and $t_{0.84}$ are the times when $\frac{C}{C_{0}}$ reaches 0.16, 0.5, and 0.84, respectively. C is the concentration of the $Br^{-1}$ in the outflow liquid, while $C_{0}$ is 0.1 mol/L.

From the longitudinal migration coefficients obtained under different F-T conditions, movements of heavy metal migration in soil can be obtained. When considered as a microcosm of the actual situation, this experiment can thus be used to predict the diffusion trend of heavy metals in the environment.

#### 2.3.3. X-ray Diffraction Analysis

A crystal is composed of a unit cell of atoms that are regularly arranged. The distance between these regularly arranged atoms is of the same order of magnitude as the wavelength of the incident X-ray in X-ray diffraction (XRD) analysis. Thus, the X-rays scattered by different atoms interfere with each other and produce strong X-ray diffraction patterns in specific directions. The orientation and intensity of the diffraction lines in the space are thus closely related to the crystal structure, as diffraction patterns produced by each crystal reflect the distribution of atoms inside that crystal. XRD tests on soil at various stages can therefore be used to effectively analyze its mineral composition.

The samples were crushed after the freeze–thaw cycles were completed and sifted through 200 mesh before being put into the dryer to dry at 100 °C for 24 h. After the samples were dried, the electron microscopy center of Chongqing University conducted the required XRD test using a D/MAX∙2500∙X-ray diffractometer to obtain details of the kinds of crystals present in the soil samples based on their X-ray diffraction patterns. The technical indexes of the instrument are shown in Table 4. Jade 6.5 was then used to analyze the composition of the samples. Before the peaks were formally identified, however, to offset the detection error of powder materials, the error of the light used in the experimental instrument, and the influence of test accuracy, the obtained diffraction pattern was processed to deduct background values. Automatic peak search, manual peak search, and PDF card comparison were then applied to match the phase.

Freeze–thaw cycles change the pore structure of the soil, which tends to promote carbonization and similar effects that can change the pH of the soil. As pH is one of the factors that affects the solidification products of heavy metals, XRD tests can help explore whether freezing and thawing reduces various HM S/S products.

## 3. Result

### 3.1. Mechanical Properties

#### 3.1.1. Stress–Strain Relationship

**Figure 5:**
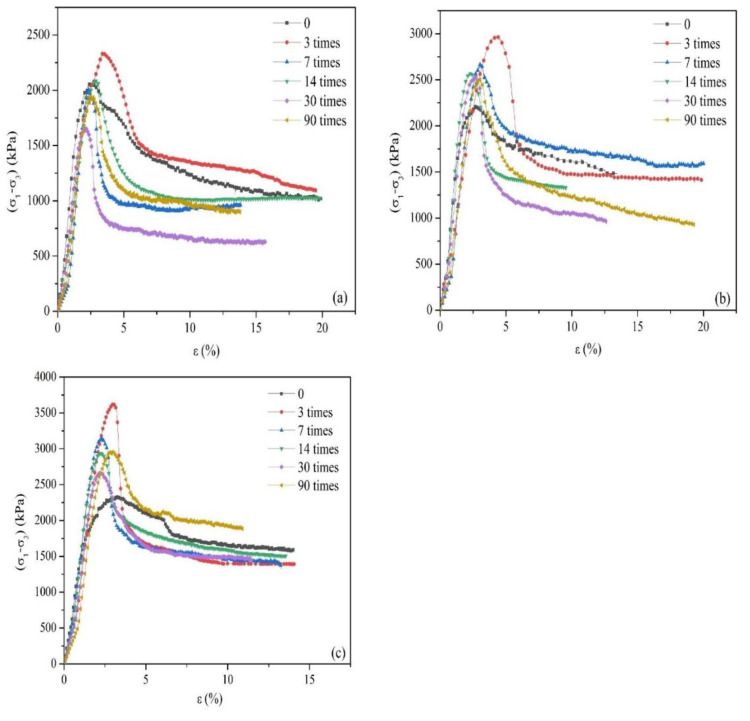
Stress–stain relationships of stabilized/solidified lead-zinc-cadmium composite heavy metal-contaminated soil under three confining stresses: (**a**) 100, (**b**) 200, and (**c**) 300 kPa.

Figure 5 shows the stress–strain relationships of stabilized/solidified composite heavy metal-contaminated soil after various F-T cycles (0, 3, 7, 14, 30, and 90 times) under the selected confining stresses (100, 200, and 300 kPa), as determined using the triaxial compression tests.

The stress–strain curve of each soil shows the peak stress, which indicates that the stress–strain curve of the stabilized/solidified composite heavy metal-contaminated soil is a stress-softening type. The range of residual stress decreases with the increase of confining pressure, as the compactness of soil increases with the increase of confining pressure. The freeze–thaw cycle thus has less effect on soil with high compactness.

Under the action of the short-term F-T cycles (3, 7, and 14 times), the position relationships of stress and strain under various dynamic and capacity conditions after the peak stress is different for each confining pressure, which indicates that the initial compactness of soil has the most significant influence on the effects of the short-term freezing–thawing cycle.

For long-term F-T cycles, Figure 5 shows that after the soil stress–strain curve exceeds the peak value, the curve under 90 freezing–thawing cycles is higher than that under 30 freezing–thawing cycles for all conditions, indicating that under the same strain conditions, soil after 90 freezing–thawing cycles will have greater strength than after 30 freezing–thawing cycles. If the number of freeze–thaw cycles is too large, however, the soil is broken up and redistributed, and the compactness and strength of the soil will change.

#### 3.1.2. Triaxial Compressive Strength

**Figure 6:**
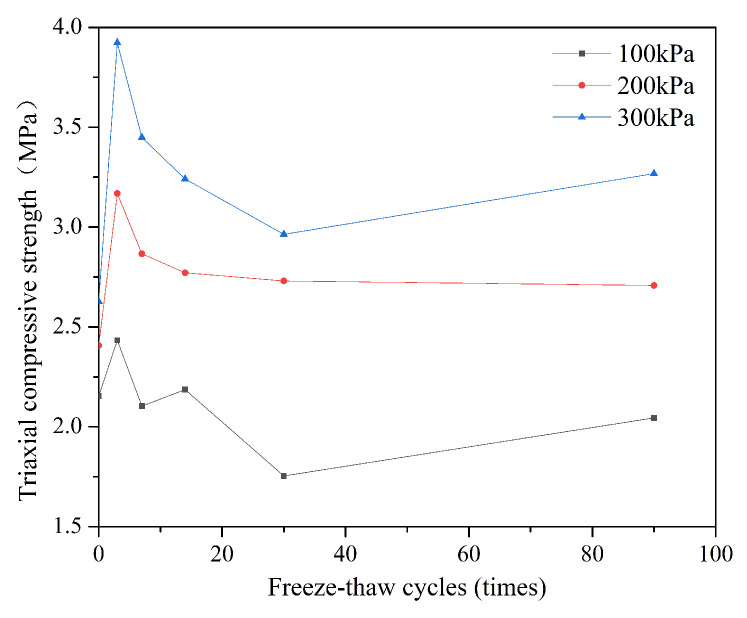
The variation of triaxial compressive strength in S/S lead-zinc-cadmium composite HM soil with different F-T cycles under three different confining stresses.

The value of triaxial compressive strength in each case is the maximum principal stress minus the minimum principal stress. Figure 6 shows the variation in triaxial compressive strength of S/S lead-zinc-cadmium composite HM soils with freeze–thaw cycles under three confining stresses (100, 200, and 300 kPa).

This indicates that larger confining pressures create larger triaxial compressive strengths, as the larger the confining pressure, the smaller the spacing, and the higher the compactness of the skeleton particles, and these factors improve the properties of the soil to some extent. As shown in Figure 6, the height of the line for 300 kPa is the highest, with the next highest being 200 kPa, and the last 100 kPa.

The tendency of each curve in Figure 6 is similar, however. Within 3 freeze–thaw cycles, the triaxial compressive strength of soil increases rapidly, while from 3 freeze–thaw cycles to 30 cycles, the triaxial compressive strengths decrease as the number of cycles increases. After 30 freeze–thaw cycles, the soil triaxial compressive strengths tend to become stable or to slightly increase. This occurs as when the number of freeze–thaw cycles is small, damage to soil is not the main influencing factor, and the water migration during the freeze–thaw action promotes the hydration of binders and improves the strength of the soil. The soil expands and shrinks continuously with the action of these early freeze–thaw cycles, and when the soil reaches the bearing range, cracks occur at the weakest parts of the soil, and then extend, resulting in a decrease in strength. The reason for the increase or stabilization of the triaxial compressive strength later may be that the soil redistributes as the freeze–thaw cycles continue, refilling previously formed large-diameter pores, which slows the ongoing accumulation of cracks, and helps increase strength. The effects of freeze–thaw cycles on soil properties may also decrease with the increase in the number of such cycles [72].

Other studies have shown that the sensitivity of soil mass is strongest at the initial stage of freeze–thaw cycles, and that it tends to be stable after additional freeze–thaw cycles.

#### 3.1.3. Indicators of Shear Strength

**Figure 7:**
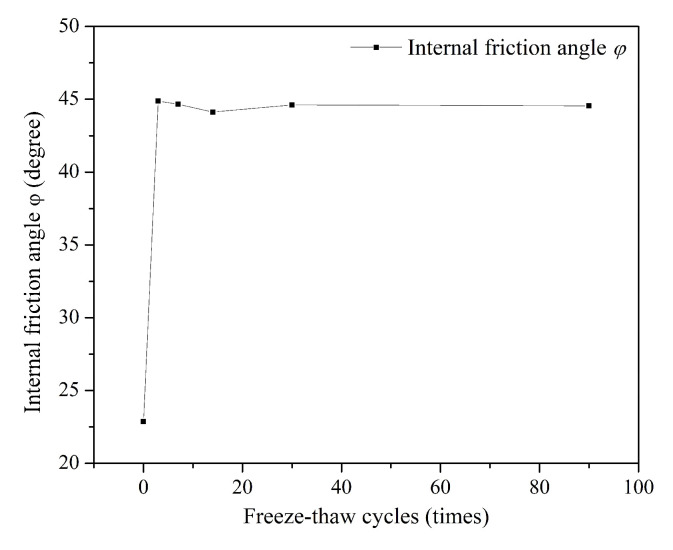
Variation of internal friction angle, φ, S/S lead-zinc-cadmium composite HM-contaminated soil with F-T cycles.

Figure 7 shows the variations in internal friction angle (φ) in S/S lead-zinc-cadmium composite HM-contaminated soils with various F-T cycles. The internal friction angle (φ) first sharply increases, then significantly decreases, finally increasing slightly. Increases in the internal friction angle of the S/S composite-contaminated soils may occur for two reasons. The first is the hydration products of cement, lime, and fly ash, which are likely to fill some pores in the soil, and this action is often sufficient to offset the negative effects of small freeze–thaw cycles. The second is that the F-T cycle enlarges the contact range between water and the three binders, improving the hydration of the binders and increasing the hydration products, which to some extent changes the roughness of the particle surface, resulting in an increase of the internal friction angle (φ) of the soil. As the number of F-T cycles continues to increase, the negative effects caused by F-T cycles dominate the soil state, and the increasing porosity ratio results in a decrease of the internal friction angle (φ). However, when the surrounding pore stresses caused by the F-T cycles match the ultimate strength of the soil, the soil particles are redistributed and are broken and the surface is gradually smoothed, which has an adverse effect on the growth of the internal friction angle (φ) under longer F-T cycles. The effect of freeze–thaw cycles on the soil internal friction angle (φ) thus becomes smaller under the combined action of porosity ratio and particle shape.

**Figure 8:**
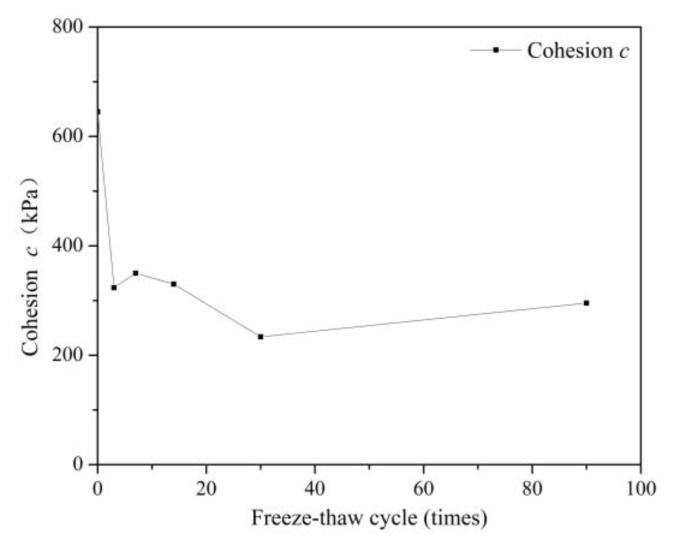
Variation of cohesion, C, of S/S lead-zinc-cadmium composite HM-contaminated soil with F-T cycles.

Figure 8 shows the variation of cohesion (C) of S/S lead-zinc-cadmium composite HM-contaminated soil with various F-T cycles: the change tendency of cohesion (C) is the opposite of that of the internal friction angle (φ). The size of cohesion is affected by the larger contact area between soil particles and is thus related to the cementing ability between particles. In the S/S HM-contaminated soil, the strongest cementing ability is undoubtedly within the mineral colloids that exist in the soil. The damage to the initial specimen under the action of early F-T cycles is thus more harmful to the cohesive force of the soil, and small cracks may lead to a loss of cohesion between soil particles. At the early stage of F-T, the resulting cracks reduce the contact area, resulting in a sharp drop in cohesion. Later, as active oxides in fly ash continue to react with cement hydration products, the quantity of colloids generated increases, which tends to increase the cohesive force of the soil. However, this impact is small, and under continued F-T, the cementing ability produced by the colloid is offset. Later, as the particles in the soil reach the ultimate strain, the ongoing F-T cycles play a positive role in redistributing the soil particles. However, the freeze–thaw cycle has a negative effect on cohesion force overall: after 90 freeze–thaw cycles, the cohesion of the soil is less than half of that of soil that has not undergone any F-T cycles.

### 3.2. Leachability

**Figure 9:**
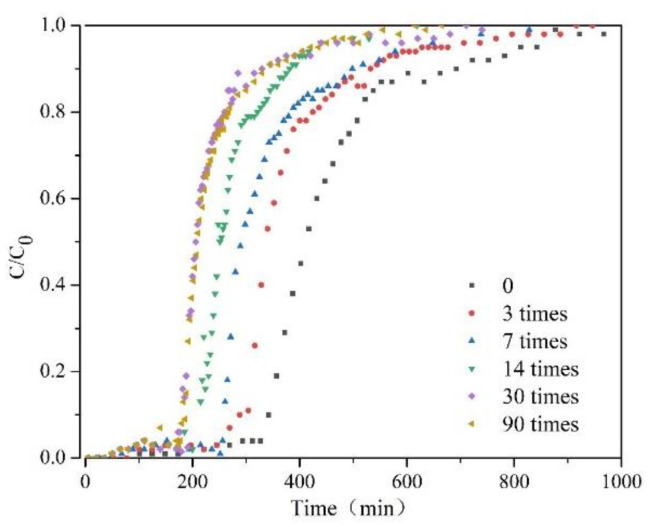
Breakthrough curves of $Br^{-1}$ of S/S lead-zinc-cadmium composite HM-contaminated soil subjected to six kinds of F-T cycles: (1) 0, (2) 3, (3) 7, (4) 14, (5) 30, and (6) 90 times.

Figure 9 shows the variation in $\frac{C}{C_{0}}$ of the flow of the liquid with time under different F-T cycles (0, 3, 7, 14, 30, and 90 times). The shape of the breakthrough curve of S/S lead-zinc-cadmium composite HM-contaminated soil without F-T cycles is centrosymmetric; however, the shapes of soils subject to F-T cycles all slide to the left. This shows that the non-equilibrium state of solute migration occurs in those soils subjected to freezing and thawing cycles. This non-equilibrium state often appears in samples with a high flow rate, and based on this phenomenon, it can be speculated that the rate of bromide ions passing through F-T soils is higher than that of those passing through non-freeze–thaw soil, where the peristaltic pump pumps the potassium bromide solution at the same speed. In the earliest F-T cycles, the average pore size of soil increases with each increase in F-T cycle number, resulting in faster movement of ions in the soil. As F-T cycles continue to increase, however, the skeleton particles in the soil mass are destroyed, which causes a small-aperture number increase, and makes the movement of ions in the soils more rapid.

**Figure 10:**
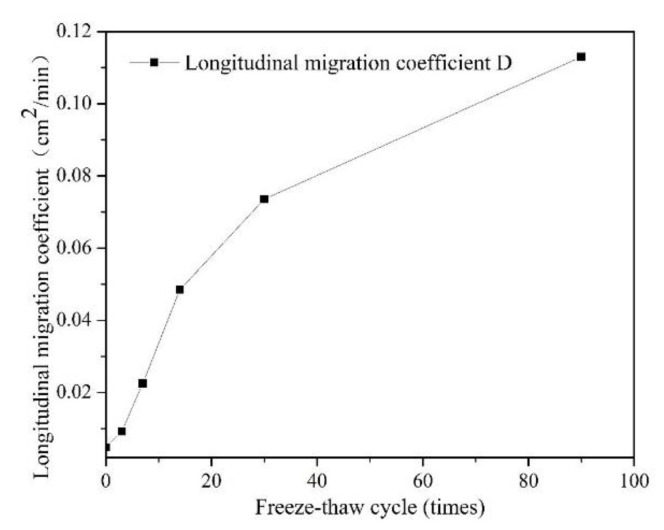
Variation of longitudinal migration coefficient (D) of $Br^{-1}$ in S/S lead-zinc-cadmium composite HM-contaminated soil with F-T cycles.

Table 5 and Figure 10 show the variation in longitudinal migration coefficient, D, in S/S lead-zinc-cadmium composite HM-contaminated soil with F-T cycles. The longitudinal migration coefficient (D) increases with the increase in F-T cycles. This indicates that heavy metals are more likely to be removed from the soil. It can thus be inferred that the F-T cycle increases the possibility of heavy metals with relatively stable forms transforming into exchangeable states, which further indicates that the F-T cycles reduce S/S effects on HM [73]. When the number of F-T cycles is too big, the third stage of the F-T cycle is reached. Based on the conditions of the destruction of the soil skeleton in the early stage, the continuation of the F-T cycle leads to the redistribution of the soil, which reduces the average pore size of the soil and increases the volume of both micropores and mesopores [74]. This may explain the increasing trend seen in both soil strength and permeability after freezing and thawing 14 times. As part of process of solidification and stabilization of heavy metal soils, it is thus necessary to consider the influence of F-T cycle conditions. According to Darcy’s law, the longitudinal migration coefficient is proportional to the square of the soil permeability coefficient. The permeability coefficient of soil after 90 F-T cycles can thus reach 4.81 times that of soil without F-T cycles.

### 3.3. Chemical Composition

**Figure 11:**
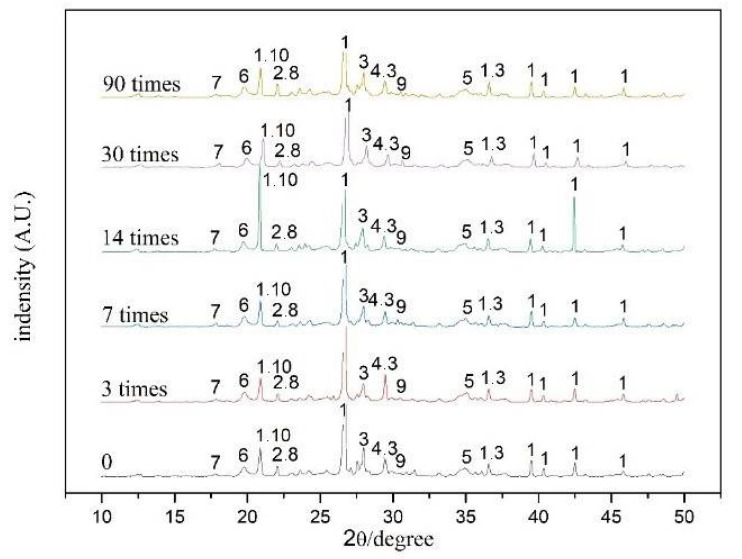
XRD patterns of the S/S lead-zinc-cadmium composite HM-contaminated soil under six kinds of F-T cycles: 0, 3, 7, 14, 30, and 90 times.

**Figure 12:**
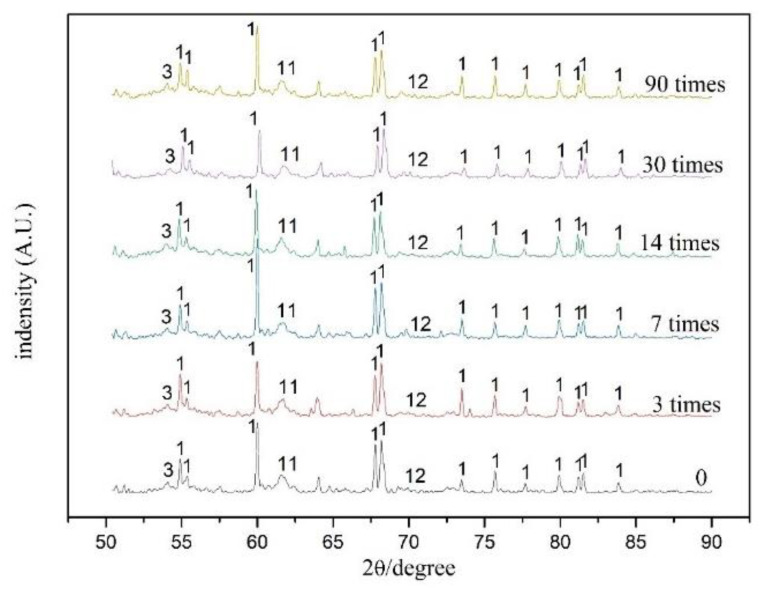
1‚SiO_2_, 2—Anorthite, 3—CSH, 4—CaZnSiO_4_, 5—Pb(OH)_2_, 6—CAH, 7—Cd(OH)_2_, 8—Clinohedrite, 9—Erionite, 10—Gismondine, 11—Zn(OH)_2_, 12‚Ca(OH)_2_.

Figure 11 and Figure 12 show the diffraction patterns of S/S artificial lead-zinc-cadmium composite HM-contaminated soil subjected to different F-T cycles (0, 3, 7, 14, 30, and 90 times). Across all samples, SiO_2_, calcium silicate hydrate gel (CSH), calcium aluminum silicate gel (CAH), Pb(OH)_2_, Zn(OH)_2_, CaZnSiO_4_, Cd(OH)_2_, Clinohedrite, Erionite, Gismondine, and Anorthite were mainly detected. These results are consistent with previous studied types of hydration products from cement, lime, and fly ash, and the main curing mechanism of heavy metals lead, zinc, and cadmium.

Of the three binders themselves, cement generates CSH, CASH, and Ca(OH)_2_ after hydration, while lime ripening increases Ca(OH)_2_ content. Various active substances, such as SiO_2_ and Al_2_O_3_ in fly ash, then convert part of this Ca(OH)_2_ into CSH and CAH.

For reactions involving heavy metals, heavy metal may precipitate with the hydration products to form Pb(OH)_2_, Zn(OH)_2_, and Cd(OH)_2_, displacing the ions in the colloid (CaZnSiO_4_, Anorthite, Clinohedrite, Erionite, and Gismondine) or becoming physically enveloped [58,64]. Lead ions first undergo a replacement reaction with calcium ions; then, when the content exceeds the required replacement amount of calcium ions, it further binds with the mineral lattice of hydration products [47,64]. Zinc reacts with cement hydration products to precipitate into calcium zincate, which wraps cement and impedes the continuation of the hydration reaction [75]. When cadmium, zinc, and lead are stabilized/solidified by cement, both chemical reactions and physical encapsulation thus occur [76].

To generate the small waveform displayed, the highest intensity portion of the data processing is not shown in Figure 11, as the particle effect of the sample resulted in a high peak strength of one crystal face under the 14-time F-T cycle condition.

The same kinds of substances were detected in soils subjected to different F-T cycles as in soils not subjected to F-T cycles, indicating that the F-T cycle has no chemical effect on the hydrolytic hydration of additives, or the S/S form of heavy metals.

## 4. Discussion

With regard to the mechanical properties of soil, the influence of the F-T cycle is mostly positive, as shown in Figure 5, Figure 6, Figure 7 and Figure 8. Compared with non-freeze–thaw (NF-T) soil, the values of triaxial compressive strength, cohesion, and internal friction angle in F-T soil are generally increased at various ranges, while only a small part of the soil shows a slight decrease.

Based on the data obtained in these tests, only when the confining pressure is 100 kPa is the triaxial compressive strength of the soil after F-T for 30 and 60 days decreased compared with that of the NF-T soil, becoming about 0.8 and 0.95 of the triaxial compressive strength of the NF-T soil respectively, in each case. When the confining pressure is 200 or 300 kPa, the triaxial compressive strength of the F-T soil is higher than that of the NF-T soil, as increasing the confining pressure of soil causes the soil particles to become more-dense, and thus the long-term F-T cycle has no deterioration effect at the confining pressures of 200 and 300 kPa. In areas with relatively loose soils, more attention should, however, be paid to the effects of long-term F-T effects on the properties of HM soils, as F-T cycles are likely to degrade soil strength over time.

The data shows that after three F-T cycles, the cohesion of S/S HM-contaminated soil increased the most, reaching three times that of the NF-T soil. In contrast, the cohesion of the soil after 90 freeze–thaw cycles was reduced to 45.7% of that of soil that had not undergone freeze–thaw cycles. Such opposing changes will affect the shear strength of the soil in practice, and in actual construction projects, it is thus necessary to pay attention to whether the freeze–thaw cycle will negatively affect the nature of a given soil to enable timely remedial measures to be applied.

Data on soil column leaching shows that the permeability of the soil increases as the number of F-T cycles increases. The longitudinal migration coefficient of the soil after 90 F-T cycles was 23 times greater than that of soil without any F-T cycles. S/S technology is not a pollution reduction technology, but rather a risk control technology. As the change in permeability of soil has a direct impact on the heavy metal solidification effect, in seasonal frozen soil areas during S/S of HMs, it is likely to be necessary to modify the curing conditions based on the results of long-term F-T studies to meet the requirements of relevant environmental regulations.

## 5. Conclusions

Based on the results of the triaxial compression tests, soil column leaching tests, and X-ray diffraction analysis tests on samples after 0, 3, 7, 14, 30, and 90 F-T cycles, the effect of long-term F-T cycle conditions on the mechanical properties, environmental influence, and the micro-phenomena of S/S lead-zinc-cadmium composite HM-contaminated soils was assessed. The following conclusions were thus drawn.

Variations in the triaxial compressive strength and cohesion of the S/S lead-zinc-cadmium composite HM-contaminated soil under most F-T cycles were similar. The changes in both factors first appeared to rise, then declined, and finally gradually stabilized. However, the freeze–thaw cycle had a negative effect on the strength of the soil overall, while the internal friction angle behaved more positively overall. The F-T cycles caused a violent decrease in cohesion initially, and although this recovered slightly in the end, a negative effect was seen overall. The action law of long-term F-T cycles is different from that of short-term F-T cycles: compared with short-term F-T cycles, several soil properties increase in stable stages after long-term F-T cycles based on triaxial compressive strength, cohesion, and internal friction angle of the soil.

The leachability of the S/S lead-zinc-cadmium composite HM-contaminated soil with F-T cycles always increases. The permeability coefficient of the soil after 90 F-T cycles was 3.81 times greater than that of the soil without F-T cycles. In the design of S/S, the F-T cycle must thus always be considered.

Under long-term F-T cycles, the chemical composition of the S/S HM-contaminated soil does not change as compared to under short-term F-T cycles. As compared with a single heavy metal contamination map, however, the products of composite heavy metal-contaminated soil are more complex, and there may also be further complex substances that are too similar in crystal structure to be correctly identified by XRD.

## Figures and Tables

**Table 1:** Basic physical properties of undisturbed soil samples.

Physical Property Index	Liquid Limit/%	Plastic Limit/%	Plasticity Index (I_p_)	Optimum Moisture Content/%	Maximum Dry Density/g/cm^3^
Soil sample	27.25	12.3	14.95	11.78	1.91

**Table 2:** Main chemical components of undisturbed soil.

Chemical Components	CaO	MgO	SiO_2_	Al_2_O_3_	Fe_2_O_3_	TiO_3_	K_2_O	ZnO	CdO	PbO	Other
Contents (%)	4.06	3.42	63.98	17.71	5.48	0.79	3.36	ND	ND	ND	1.2

Note: ND means not detected.

**Table 3:** Chemical components of the three binders.

Chemical Components	CaO	MgO	SiO_2_	Al_2_O_3_	Fe_2_O_3_	TiO_3_	K_2_O	SO_3_	ZnO	CdO	Others
Cement (%)	53.41	3.60	26.69	7.44	2.64	0.37	1.32	4.04	0.02	0.02	0.48
Lime (%)	88.73	4.79	3.88	1.09	0.73	0.10	0.23	0.40	ND	ND	0.16
Fly ash (%)	4.63	0.75	58.62	26.64	4.32	1.19	2.11	0.61	0.02	ND	1.08

**Table 4:** Technical index, D/MAX X-ray diffractometer.

Maximum Power	18 kW
Tube voltage	20–60 kV (1 kV/step)
Tube current	10–300 mA (1 mA/step)
Stability	Within ± 0.01%
Minimum stepping angle	1°/1000
Minimum step	1°/10,000
Set repeatability	1°/10,000
Silt	Automatically variable slit

**Table 5:** Longitudinal migration coefficient (D) under different F-T cycles.

F-T Cycles	Soil Column Length (cm)	Pore Water Velocity (cm/min)	$T_{0.16}$ (min)	$T_{0.5}$ (min)	$T_{0.84}$ (min)	D $\left({\mathrm{cm}}^{2}/\min\right)$
0	20	0.0222	352	409.5	532	0.004874
3	20	0.0337	308	337.5	456	0.009231
7	20	0.0427	262	290.5	431.5	0.022540
14	20	0.0829	224	251	343	0.045466
30	20	0.1284	186	206	265	0.073590
90	20	0.1389	190	209	295	0.112985

## Data Availability

The data that support the findings of this study are available from the corresponding author, upon reasonable request.
